# Prognostic factors related to neurological disability after the implementation of the first stroke unit in a remote region of Brazil

**DOI:** 10.1055/s-0046-1827210

**Published:** 2026-08-31

**Authors:** Ana Rosa Ribeiro Fonseca, Allex Jardim da Fonseca, Lívia Maria da Silva Martins, Caio Cayres de Queiroz, Joab Ferreira de Oliveira Júnior, Ananda de Melo Albuquerque, Kelven Henrique Silva de Sousa, Raquel Gaio de Matos, Gustavo Procópio Silva, Ruan Lucas de Almeida Mangueira, André Machado de Siqueira

**Affiliations:** 1 Instituto Oswaldo Cruz/Fiocruz, Programa de Pós-Graduação em Medicina Tropical, Rio de Janeiro RJ, Brazil.; 2 Universidade Federal de Roraima, Centro de Ciências da Saúde, Departamento de Medicina, Boa Vista RO, Brazil.

**Keywords:** Stroke Rehabilitation, Stroke, Thrombolytic Therapy, Statistics on Sequelae and Disability

## Abstract

**Background:**

Stroke is the main public health problem in Brazil.

**Objective:**

To evaluate the incidence of moderate-to-severe disability in patients admitted to the Stroke Unit in Roraima (2024), and estimate the impact of Stroke Unit implementation on neurological outcomes compared with historical data.

**Methods:**

This hospital-based observational cohort study assessed factors associated with moderate-to-severe disability in patients admitted to the Stroke Unit. Clinical and sociodemographic data were collected using a semistructured questionnaire. Participants were evaluated daily until discharge or death using the National Institutes of Health stroke scale (NIHSS).

**Results:**

A total of 400 patients was included. Mean age was 64.2 ± 15.8 years, and 52% of patients were male. Most cases were of ischemic stroke (82.2%), followed by wake-up stroke (13%), transient ischemic attack (TIA: 10%), and hemorrhagic stroke (1%). Systemic arterial hypertension (SAH: 57.1%), and diabetes (33.1%) were the most frequent comorbidities. Nearly 80% of patients were discharged, and the mortality rate was 3.7%. According to the modified Rankin Scale (mRS), moderate-to-severe disability at day 90 was associated with age > 70 years (RR = 1.45; 95% CI = 1.02–4.53), previous stroke (RR = 1.35; 95% CI = 1.12–3.42), and structural heart disease (RR = 1.80; 95% CI = 1.23–3.28). Thrombolytic therapy was performed in 12.5% of cases and reduced moderate-to-severe sequelae by 50%.

**Conclusion:**

Stroke Unit implementation represented a significant advance in stroke care in Roraima, reducing sequelae severity and mortality, including among older patients. However, access to thrombolytic therapy remains limited due to delayed hospital admission.

## INTRODUCTION

Stroke is one of, if not the main public health problem in Brazil and worldwide. It is characterized by marked inequalities in morbidity and mortality across populations, driven by sociodemographic and clinical determinants that affect both the incidence of stroke and access to treatment. 1 Unlike global trends, stroke mortality has risen in Brazil over the past 5 years. 2

Although Stroke Units were conceived in the 1950s as organized health systems for managing stroke, it took almost half a century for them to be effectively implemented. Since then, numerous clinical studies have provided robust evidence of the benefits of multidisciplinary rehabilitation and of the prevention of stroke complications. 3 4 5 However, thrombolytic therapy became established in 1995 as the strategy with the greatest impact on the natural history of stroke. The pivotal study conducted by the National Institute of Neurologic Diseases in 1995 demonstrated a significant reduction in disability and neurological deficits with thrombolytic therapy using alteplase. 6

Despite this, the integration of these therapeutic strategies into healthcare systems in Brazil and worldwide has been slow. 7 This is partly due to resistance to changing the treatment paradigm, but mainly due to the relative complexity of the investments required to install and disseminate these units. Indeed, even today, few stroke victims have access to a Stroke Unit, even in many more developed countries. 8 In Brazil, the situation is no different. Ordinance No. 664/2012 standardized clinical protocols and therapeutic guidelines for stroke in Brazil, including thrombolytic therapy, and listed the qualification criteria for implementing a Stroke Unit in authorized hospitals. Although this standardization has been in effect for over 10 years, implementing it remains a persistent challenge in Brazil, particularly in the country's most isolated regions. 7 9

The state of Roraima, located in the Northern Amazon, is the smallest and most isolated in Brazil. Until recently, stroke care was provided exclusively in standard wards, with adverse consequences for the affected population. In 2018, a prospective hospital-based study found that most stroke patients in Roraima were admitted with moderate neurological deficits on the National Institutes of Health stroke scale (NIHSS: 65%) and within the appropriate time window for thrombolytic therapy. However, because disease-modifying treatments and systematic, organized support (Stroke Unit) were unavailable at the time, the study reported a high incidence of neurological disability and a significant social impact on survivors. 10 This reality began to change in November 2022, following the implementation of the first Stroke Unit in the state of Roraima and the initiation of thrombolytic therapy.

The objective of this study is to evaluate the incidence of moderate-to-severe neurological disability among patients admitted to the Stroke Unit in Roraima in 2024 and to estimate its impact on the neurological outcomes of surviving patients, using historical data for comparison.

## METHODS

### Study design

This is a prospective observational cohort study designed to assess functional disability on day 90 among patients admitted to the Stroke Unit at the Hospital Geral de Roraima (HGR) in Brazil in 2024.

### Population and scenario

The HGR Stroke Unit is a type 2 unit accredited by the Ministry of Health and the Brazilian Society of Cerebrovascular Diseases. It is the only one operating in Roraima, a Brazilian state in the Northern region. It was authorized by the Ministry of Health in 2022. The unit has 13 neurointensive care beds, including one dedicated exclusively to thrombolysis, and a multidisciplinary team comprising neurologists, general practitioners, and other specialists trained and qualified for stroke center care.

### Research procedures

Upon admission to the HGR Stroke Unit, all patients were approached by the team and invited to participate in the study. When participation was clinically impossible, their legal representatives were approached. If accepted, a consent form was signed, and only then were any study procedures performed.

The inclusion criteria were patients admitted to the Stroke Unit with a diagnosis of ischemic or hemorrhagic stroke, transient ischemic attack (TIA), or cerebral venous thrombosis. The exclusion criteria were patients under 18-years-old and those who did not consent or sign the informed consent form.

All patients with recent neurological deficits underwent CT scans upon admission. If the initial CT scan was normal, a second was performed after 48 hours to confirm the diagnosis of ischemic stroke by documenting an ischemic brain lesion. If no lesion was identified on the follow-up CT scan (48 hours), brain magnetic resonance imaging (MRI) and cerebral magnetic resonance angiography were performed to characterize the condition and differentiate between TIA and stroke mimics. Cases of wake-up stroke were not eligible for thrombolysis. Cases of hemorrhagic stroke were preferentially referred to another Intensive Care Unit (ICU), under the care of the neurosurgery department.

Upon admission, sociodemographic and clinical data were recorded as explanatory variables, with a focus on prior comorbidities and stroke characteristics. These data were obtained through interviews and clinical evaluations conducted by the research team, as well as from medical records and additional tests, when necessary. Therapeutic strategies and complications of the clinical condition were recorded daily until hospital discharge, transfer, or death. At 90 days after stroke onset, participants were assessed for disability using the modified Rankin Scale (mRS), marking the end of study participation. For survivors discharged from the hospital, the 90-day assessment was conducted via a standardized telephone interview or an outpatient clinical review. Team members responsible for evaluating the NIHSS and mRS were adequately trained.

All patients admitted into the Stroke Unit were included in the descriptive analysis to characterize the clinical and sociodemographic profile. Only patients diagnosed with ischemic stroke were included in the univariate and multivariate risk analyses, to avoid sample heterogeneity. The primary outcome was the incidence of moderate-to-severe disabilities, defined as an mRS score >2 on day 90. Secondary outcomes were mortality and NIHSS score at discharge. There was no intervention. Data were recorded simultaneously to treatment.

### Sample size and selection

The sample size calculation was performed using an estimated incidence of 40% for the outcome (moderate to severe neurological disability). Based on a similar study, 11 assuming a 95% confidence interval (CI) and an acceptable error of 5%. The initial sample size target was, therefore, 363 participants. To account for the risk of data or sample loss, the target was increased by 10%, resulting in a final target of 399 participants.

The sampling method was systematic. All patients (or their legal representatives) were invited to participate and were consecutively included from the start date (January 2024) until the target sample size was reached, without patient selection.

### Data analysis methods

Clinical, demographic, and socioeconomic data were collected for all participants, including stroke characteristics, comorbidities, age, ethnicity/race, marital status, origin, clinical and functional status at admission, treatment, and preventive strategies. These data were also used as explanatory variables in the risk analysis, which included only participants with ischemic stroke.

Descriptive statistics were computed, including frequency distributions for categorical variables and means with standard deviations (SDs) or medians with interquartile ranges (IQRs) for continuous variables with normal and nonnormal distributions, respectively. The incidence of the outcome variables, along with their 95% CI, was estimated using binomial distribution. To compare sample means, Student's t-test was used for variables with normal distributions and homogeneous sample variances. When this was not possible, the Mann-Whitney test was used.

The Chi-squared test was used to compare proportions across categorical variables. Correlations were quantified using relative risk (RR) and 95% CI in bivariate analysis. Variables that showed a correlation with the disability outcome in the bivariate analysis were reanalyzed in the multivariate analysis to control for confounding variables, yielding an adjusted relative risk (aRR). Multivariate analysis was performed using Poisson's regression with robust variance, and the criterion for variable selection was a *p* -value < 0.10 in the bivariate analysis. All statistical analyses were performed using R software (R Foundation for Statistical Computing), version 4.3.1. The significance level was set at 5%.

### Ethical aspects

The study was evaluated and approved by the Institution's Board of Directors and the UFRR's independent Human Research Ethics Committee. All research documents were coded to remove personal identifiers and protect confidentiality. No intervention was conducted, and all patients were informed that nonparticipation would not affect their treatment.

## RESULTS

**Fig. 1:**
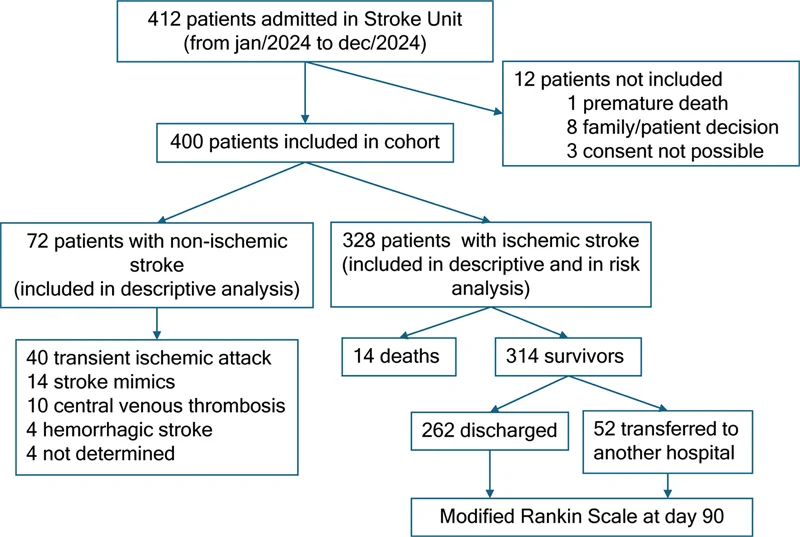
Flowchart for inclusion and follow-up of participants.

During the study inclusion period (January 12, 2024, to December 26, 2024), 412 patients were admitted to the Stroke Unit. Of these, 12 were excluded due to premature death (n = 1), decision not to participate (n = 8), and inability to obtain consent (n = 3). As such, a total of 400 patients were included in the study ( Figure 1 ).

The mean age was 64.2 ± 15.8 years, and 52% of the sample was male. Approximately 3% were indigenous, and 8% were foreign nationals. The most common comorbidities were systemic arterial hypertension (SAH: 57.1%), diabetes mellitus (33.1%), and dyslipidemia (15.0%). More than a third of patients had a prior stroke (37.7%), with cortical topography as the most prevalent (13.3%). Regarding prior medication use, 14.6% used antiplatelet agents, and 5.8% used anticoagulants. Among female patients, only 2.5% used oral contraceptives. Table 1 presents the clinical and sociodemographic data.

**Table 1:** Sociodemographic and clinical characteristics of the sample (n = 400)

Sociodemographic characteristics	Mean ± SD	n (%)
**Age, years**	64.2 ± 15.8	
< 50 > 60		79 (19.8%) 270 (67.7%)
**Female gender**		192 (48%)
**Self-declared ethnicity**		
Mixed-race White Black Indigenous		278 (69.5%) 76 (19.0%) 33 (8.3%) 13 (3.2%)
**Nationality** Brazilian Venezuelan/Guyanese		367 (91.8%) 33 (8.2%)
**Transport to the hospital**		
Private transport Regular public transport Ambulance		197 (49.2%) 40 (10.0%) 163 (40.8%)
**Previous clinical characteristics**		
**Previous morbid conditions**		
SAH Diabetes mellitus Dyslipidemia Use of oral contraceptives (women; n = 192) Previous use of antiplatelet agents Previous use of oral anticoagulants		288 (57.1%) 132 (33.1%) 60 (15.0%) 10 (2.5%) 58 (14.6%) 23 (5.8%)
**Previous stroke**		151 (37.7%)
Cortical Subcortical Brainstem		53 (13.3%) 90 (22.6%) 8 (2.0%)
**Stroke characteristics**		
**Symptom onset to hospital admission time**		
All patients (n = 400) < 1h < 3h < 4.5h	12.0 ± 21.1	19 (4.7%) 54 (13.5%) 60 (15.0%)
Foreign national (n = 33) < 4.5h	18.4 ± 36.5	2 (6.0%)
Private transport (n = 197) < 4.5h	9.5 ± 28.3	39 (19.7%)
Ambulance transport (n = 163) < 4.5h	15.2 ± 29.1	21 (12.8%)
**Type of stroke**		
Ischemic TIA Hemorrhagic Stroke mimics Cerebral venous thrombosis Not determined Wake up stroke		327 (82.2%) 40 (10%) 4 (1%) 14 (3.5%) 10 (2.5%) 3 (0.75%) 52 (13%)
**Admission exams** Doppler ultrasound Structural heart disease/atrial fibrillation		100 (25.0%) 52 (13.1%)
**Systolic blood pressure on admission (mmHg)** < 120 > 185	156 ± 33.1	55 (13.8%) 84 (21.0%)
**NISS on admission**	10.5 ± 6.5	
Light (0–4) Moderate (5–15) Moderate to severe (16–20) Severe (> 20)		71 (19.8%) 212 (59.0%) 45 (12.5%) 31 (8.6%)
**Hospitalization characteristics**		
**Therapy instituted / maintained**		
AAS Clopidogrel Rivaroxaban Invasive ventilation Craniectomy Thrombolytic therapy		319 (80.1%) 87 (21.8%) 59 (14.8%) 28 (7.0%) 7 (1.8%) 50 (12.5%)
**Thrombolytic therapy characteristics (n = 50)**	57 ± 43.1	
Symptom onset to hospital < 1h Symptom onset to hospital < 3h Door to needle time (min) Door to needle time < 30 min Door to needle time < 60 min		16 (32.0%) 37 (74.0%) 8 (16.0%) 25 (50.0%)
**Complications**		
Hemorrhagic transformation of the ischemic area Extracranial bleeding Sepsis Pneumonia		4(1.0%) 0 12 (3.0%) 41 (10.3%)
**Hospitalization outcome**		
Hospital discharge Transfer to another hospital Death		318 (79.7%) 66 (16.6%) 15 (3.7%)
**NHISS at discharge/transfer (n = 362)**	6.3 ± 5.6	
Light (0–4) Moderate (5–15) Moderate to severe (16–20) Severe (>20)		169 (46.7%) 159 (43.9%) 24 (6.6%) 10 (2.7%)
**mRS on day 90 (ischemic stroke only, n = 328)**		
0–1 2 3–4 5–6		116 (35.5%) 105 (32.0%) 99 (30.3%) 7 (2.2%)

Abbreviations: ASA, acetylsalicylic acid; CI, confidence interval; mRS, modified Rankin Scale; NIHSS, National Institutes of Health stroke scale; TIA, transient ischemic attack; RR, relative risk; SAH, systemic arterial hypertension.

The average time from symptom onset to hospital arrival was 12 ± 21.1h, and only 15% of patients were admitted within 4.5h of symptom onset. The proportion of foreign patients arriving at the hospital quickly was lower (only 6% within 4.5h). Regarding the clinical presentation of current stroke, most cases were ischemic stroke (82.2%), followed by TIA (10%); only 1% presented with hemorrhagic stroke. Wake-up stroke was documented in 13% of the sample. Stenosis was detected by routine carotid Doppler ultrasound in 25% of the sample, and structural heart disease or atrial fibrillation was identified in 13.1% of routine echocardiogram and/or electrocardiogram examinations. Systolic blood pressure on admission greater than 185 mmHg was recorded in 21%.

Among the admission severity scale (NIHSS) scores, the most common presentation was moderate impairment (NIHSS: 5–15, 59.0%), followed by mild (NIHSS: 0–4, 19.8%). Acetylsalicylic acid (ASA) was used in 80.1% of cases, clopidogrel in 21.8%, and rivaroxaban in 14.8%. Only 55 patients (13.7%) were admitted to the Stroke Unit within 4.5 hours of symptom onset. Thrombolytic therapy was administered to 50 patients (12.5%).

**Fig. 2:**
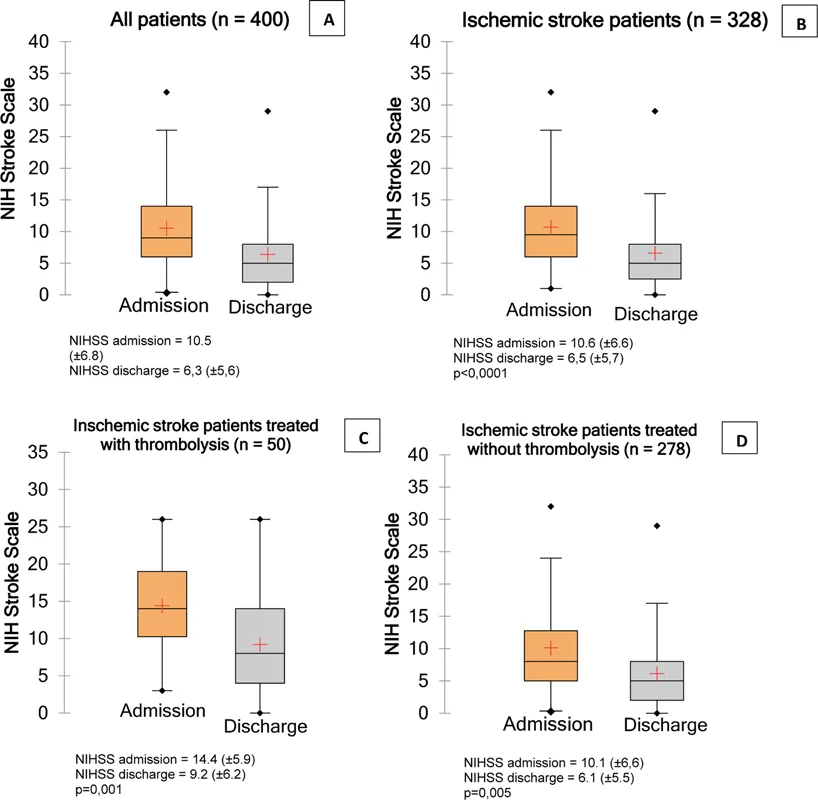
Comparison of NIHSS scores at admission and discharge. ( **A** ) All patients included. ( **B** ) Patients hospitalized for ischemic stroke. ( **C** ) Patients hospitalized for ischemic stroke and treated with thrombolysis. ( **D** ) Patients hospitalized for ischemic stroke and not treated with thrombolysis.

Across the entire sample, the incidence of hemorrhagic transformation of the ischemic area was 1%, and there were no cases of systemic bleeding. Infectious complications were the most common, with pneumonia in 10.3% and sepsis in 3% of cases. Almost 80% of patients were discharged with recovery or improvement, and 16.6% were transferred to another hospital. Only 15 patients died, corresponding to a 3.7% mortality rate. For stroke survivors, reassessment of the NIHSS revealed a higher proportion of mild clinical impairment (46.7%), followed by moderate (43.9%). Severe impairment (NIHSS > 20) was recorded in only 2.7%. Figure 2 illustrates the evolution of the NIHSS across different groups.

All ischemic stroke patients were reassessed on day 90 for functional disability, either by in-person clinical examination or standardized telephone contact. The incidence of moderate to severe disability was 32.5% (mRS > 2). Outcome data are detailed in Table 2 . In bivariate risk analysis, age had a significant impact. Being over 60-years-old increased the incidence of disability by 40%. At the 70-year cutoff, the increase in disability incidence was approximately 60%. History of stroke was also a risk factor for severe disability (RR = 1.58). Previous diabetes mellitus, SAH, or prior use of antithrombotics did not correlate with more severe disability at outcome.

**Table 2:** Assessment of risk of moderate to severe disability (mRS > 2 on day 90) among ischemic stroke patients. Boa Vista, Brasil (n = 328)

Explanatory variable	n	Incidence of moderate to severe disability (%)	*p* -value	RR (95% CI)
**Patients**	328	32.5	−	−
**Female** **Male**	149 179	29.5 34.6	0.38	0.79 (0.49–1.26) 1
**Nationality**				
Foreign Brazilian	26 302	23.1 33.1	0.29	0.60 (0.24–1.51) 1
**Ethnicity/Race**				
Mixed-race White Black Indigenous	225 63 28 12	31.5 30.8 33.6 37.5	0.43 0.38 0.42 0.011	0.94 (0.80–2.62) 0.85 (0.62–3.21) 1.14 (0.42–4.89) 1.42 (0.32–6.59)
**Age, years**				
> 50 < 50 < 60 < 70	60 268 226 127	25.0 33.9 39.5 42.4	- 0.24 0.03 0.01	1 1.54 (0.82–2.89) 1.40 (1.09–2.82) 1.62 (1.15–3.68)
**Previous medical conditions**				
SAH Diabetes mellitus Previous stroke Dyslipidemia Antiplatelet use Anticoagulant use Contraceptive use (fem)	194 108 104 48 49 16 6	36.6 34.0 40.3 33.3 34.7 32.8 16.6	0.17 0.25 0.02 0.87 0.67 0.51 0.40	1.38 (0.88–2.28) 1.28 (0.78–2.16) 1.58 (1.08–2.78) 0.94 (0.49–1.80) 0.87 (0.46–1.64) 1.46 (0.48–4.41) 0.54 (0.08–14.9)
**NIHSS on admission**				
> 4 < 4	264 64	39.0 4.7	< 0.0001	13.0 (4.39–39.27) 1
**mRS on admission**				
3–5 0–2	16 312	68.8 30.4	0.001	5.05 (1.76–14.27) 1
**Symptom onset to hospital time**				
< 1h > 1h	19 309	30.3 49.5	0.038	0.65 (0.20–0.98) 1
< 3h > 3h	41 287	29.6 51.22	0.006	0.40 (0.18–0.77) 1
< 4.5h > 4.5h	45 283	29.7 48.9	0.010	0.41 (0.25–0.83) 1
< 12h > 12h	148 180	28.3 37.1	0.09	0.68 (0.42–1.35) 1
**Systolic blood pressure on admission**				
> 180 mmHg < 120 mmHg	62 29	25.8 37.9	0.22 0.49	0.68 (0.36–1.26) 1.31 (0.60–2.84)
**Wake up stroke** No	42 286	30.9 32.5	0.84	0.97 (0.72–1.30) 1
**Carotid stenosis** No	85 243	32.4 31.7	0.85	1.09 (0.71–1.43) 1
Heart disease/atrial fibrillation No	40 321	43.5 29.7	0.015	1.75 (1.12–2.88) 1
**Antithrombotic therapy**				
AAS Clopidogrel Rivaroxaban	272 67 37	32.3 19.4 39.8	0.87 0.017 0.03	0.81 (0.62–1.07) 0.71 (0.22–0.88) 1.32 (1.07–1.75)
**Thrombolytic therapy** No	45 283	26.3 53.3	0.001	0.53 (0.08–0.86) 1
**Pneumonia** No	34 326	53.3 28.9	0.01	1.85 (1.21–4.27) 1

Abbreviations: ASA, acetylsalicylic acid; CI, confidence interval; mRS, modified Rankin Scale; RR, relative risk; SAH, systemic arterial hypertension.

Among clinical data from the current stroke, the initial severity assessment was most strongly associated with disability at 90 days. An NIHSS score > 4 at admission increased the risk by more than 10-fold (RR = 13.0), followed by a high mRS at admission (RR = 5.05), suggesting preexisting disability. Shorter times between symptom onset and hospital arrival were a significant protective factor, with a 35% reduction for intervals less than 1 hour and a 60% reduction for intervals less than 4.5 hours.

The detection of structural heart disease and/or atrial fibrillation significantly increased the incidence of disability by 75%, and pneumonia during hospitalization increased this incidence by 85%. Detection of carotid stenosis did not correlate with a worse prognosis. The use of clopidogrel as therapy for current stroke was associated with a 30% reduction in disability (RR = 0.71). This benefit was not observed with ASA. The use of the anticoagulant rivaroxaban was associated with a 32% increase in the risk of moderate to severe disability (RR = 1.32). The most significant reduction in the incidence of stroke disability was observed with thrombolytic therapy, with a clinical benefit of approximately 50% (RR = 0.53; 95% CI = 0.08–0.86; *p* = 0.001).

Variables that correlated in the bivariate analysis were reanalyzed in a multivariate analysis ( Table 3 ). Although age over 60 years was not confirmed, age over 70 years was an independent risk factor (aRR = 1.45). Admission severity (NIHSS > 4 and mRS > 20) remained the most significant independent risk factors. Short time intervals (< 3 and < 4.5h) between symptom onset and hospital arrival were also confirmed to be protective. Initiation of clopidogrel or rivaroxaban therapy was not confirmed to be associated with the outcome. However, thrombolytic therapy was a significant protective factor for disability (aRR = 0.50, 95% CI = 0.09–0.92). Preexisting heart disease and pneumonia after hospitalization remained risk factors, increasing the risk of disability at day 90 by more than 50%.

**Table 3:** Multivariate analysis for the risk of moderate to severe disability, for patients with ischemic stroke

Explanatory variable	*p* -value	aRR (95% CI)*
Age (> 70 years)	0.046	1.45 (1.02–4.53)
Previous stroke	0.01	1.35 (1.12–3.42)
NIHSS > 4 at admission	<0.0001	10.1 (2.35–27.25)
mRS 3–5 at admission	0.002	4.80 (1.25–11.47)
Symptom onset to hospital time		
< 1h	0.087	0.87 (0.14–2.58)
< 3h	0.02	0.52 (0.22–0.83)
< 4.5h	0.010	0.50 (0.25–0.72)
< 12h	0.13	0.78 (0.52–2.75)
Heart disease/atrial fibrillation	0.012	1.80 (1.23–3.28)
Antithrombotic therapy with clopidogrel	0.065	0.85 (0.36–1.12)
Antithrombotic therapy with rivaroxaban	0.082	1.09 (0.62–3.25)
Thrombolytic therapy	0.003	0.50 (0.09–0.92)
Pneumonia in hospitalization	0.03	1.54 (1.12–6.52)

Abbreviations: aRR, adjusted relative risk; CI, confidence interval; mRS, modified Rankin Scale; NIHSS, National Institutes of Health stroke scale.

Note: *The outcome in analysis is moderate–severe sequelae, with mRS > 2 on day 90.

## DISCUSSION

This study aims to assess the incidence of functional disability and its prognostic factors among stroke patients in a previously studied region, but in a new setting: after the implementation of the first Stroke Unit in Brazil's most isolated state. This provides an opportunity to estimate the impact of systematizing Stroke Unit care by comparing it with historical data from a methodologically similar study conducted by the same group in the same region 7 years earlier.

Advanced age was identified as a major risk factor for moderate-to-severe disability, with a significantly increased risk among patients over 70. These findings align with recent studies, such as that by Peng et al., 12 which highlight that advanced age is associated with higher mortality, a higher risk of stroke recurrence, and slower functional recovery.

Although a study conducted in the same region (Roraima) in 2018 corroborates this unfavorable prognosis, the magnitude of the impact of age on stroke differed. 10 In 2018, being over 70-years-old increased the risk of severe disability by 97%, whereas in 2024 the same age cutoff increased the risk of disability by 45%. This difference suggests a possible mitigating effect of implementing the Stroke Unit on the advanced age risk factor. A similar pattern was observed for history of stroke. In 2018, this prognostic factor increased the risk of disability by 74%, whereas in the current study the increase was 35%. Although these are nonmodifiable and independent factors of poor prognosis, the systematization of care may mitigate their adverse effects. Caution is advised when comparing historical data across studies, as causality cannot be inferred. Therefore, we should interpret these comparisons as descriptive rather than causal.

In the present study, the factors most strongly correlated with severe disability at day 90 were NIHSS > 4 (aRR = 10.1) and mRS 3 to 5 (adjusted RR = 4.8) at admission. It is important to clarify that the baseline functional status (mRS at admission) refers to the preindex event condition, because only preevent status allows for a meaningful comparison with the 90-day outcome, given the inherent difficulty of accurately assessing functional status during the acute phase of the disease.

The current study reported a lower mortality rate than the previous one, conducted in Roraima before the implementation of the Stroke Unit. Although our study was not designed to directly compare mortality among patients treated inside and outside the Stroke Unit, a Brazilian study did so in São Paulo in 2013. Rocha et al. 13 reported that mortality among patients treated for ischemic stroke in general wards was 13.1%, compared with 3.5% in the Stroke Unit of the same hospital during the same period. These mortality rates are very similar to those reported in studies from our region (16.0% in 2018, treated in standard wards, and 3.7% in 2024, treated in the Stroke Unit).

A meta-analysis of nearly 7,000 patients' data confirmed that Stroke Units are associated with a 20% reduction in in-hospital mortality. 14 Although much of this reduction is attributed to the rapid administration of thrombolytic therapy, other factors are also important. In our study, this was the most effective intervention, reducing the risk of severe disability by approximately 50%. However, thrombolysis was performed in only 12.5% of patients; therefore, it is insufficient to explain such a significant reduction in mortality. This reinforces the evidence presented by Langhorne in 2021, 4 which highlights the benefits of multidisciplinary and systematic care in specialized Stroke Units, including early rehabilitation, a trained team, and prevention of complications, as the main interventions for reducing mortality.

Although the use of rivaroxaban was associated with a 21% increased risk of disability in bivariate analysis, this association was not confirmed in multivariate analysis, suggesting that the observed effect may be due to confounding. In our study, the incidence of hemorrhagic transformation of the ischemic area was very low (1%), and no cases of systemic bleeding were recorded, highlighting the safety of antithrombotic and thrombolytic therapy in this context. Rivaroxaban use is associated with structural heart disease and significant arrhythmias. In our sample, structural heart disease or prior atrial fibrillation (aRR = 1.80) was also identified as an independent determinant of worse stroke prognosis, in line with evidence from Kleinig et al. 8 The Atrial Fibrillation Rhine-Neckar Region (ARENA) study found that patients with atrial fibrillation and a history of heart disease have a significantly worse prognosis for stroke. 15

Only 13% of patients arrived at the hospital within 3 hours of symptom onset. Thrombolysis was administered to 12.5% of all patients. This suggests that the main barrier to thrombolytic therapy is ineligibility due to the time window. It should also be noted that the time window was worse for immigrants and for those dependent on ambulance transport, reinforcing the social determinants of inequity. Although Roraima has the highest proportion of indigenous people in Brazil (15% of the population), only 3% of admitted patients were indigenous, suggesting limited access, particularly due to geographical barriers, as 85% of Roraima's indigenous population lives in villages within the forest. Kleinig et al. 8 highlighted that, even in developed countries, access to a Stroke Unit remains limited by time constraints, reinforcing the need for improvements in care flow and public awareness of the signs and symptoms of stroke.

A study conducted in Ceará's countryside (a Brazilian state also marked by greater healthcare disparities) highlights significant advances in stroke care 3 years after the implementation and improvement of the Stroke Unit, including a reduction in door-to-needle time from 39.5 minutes in the first year to 17 minutes in the 3 ^rd^ year and an increase in thrombolysis rates from 10.5 to 24.1%. 16 These improvements were achieved through strategies such as team training, prehospital education, and enhanced coordination between emergency and hospital services. In contrast, Roraima faces unique challenges as a more isolated state, which likely affects resource availability, access to specialized care, and the implementation of organized stroke care lines. The state's geographical isolation and limited infrastructure may hinder rapid response times and multidisciplinary collaboration, both of which were key to the success in Ceará. This comparison underscores the importance of addressing regional disparities in healthcare access and infrastructure to improve stroke outcomes across Brazil.

In conclusion, the implementation of the Stroke Unit at the HGR marked a significant advance in stroke management in Roraima. The present study demonstrated that the Stroke Unit contributed to a historic reduction in disability severity and mortality, even among older patients, highlighting the positive impact of thrombolytic therapy, which reduced the risk of moderate-to-severe disability by 50%. However, access to thrombolytic therapy remains low due to an inappropriate time window for admission. Therefore, it is essential to continue investing in infrastructure, professional training, along with prevention and public awareness strategies to consolidate the observed benefits and expand access to treatment for populations in this remote region of the country.
